# Mental Health of the Roma Population in Spain: A Scoping Review

**DOI:** 10.1007/s40615-025-02415-8

**Published:** 2025-04-14

**Authors:** Lara Pais, Lucía del Río-Casanova, Yolanda García, Carlos Ferrás

**Affiliations:** 1 Department of Psychiatry, Medical School, University of Santiago de Compostela, Santiago, Spain; 2 Education and Social Work Faculty, University of Vigo, Ourense, Spain; 3 Human Geography Department, Geography and History Faculty, University of Santiago de Compostela, Santiago, Spain

**Keywords:** Mental health, Psychiatry, Psychology, Social work in health, Demography, Roma population

## Abstract

**Objectives:**

The Roma population in Spain faces multiple known risk factors and shows indicators of a worse mental health status than the general population. A few studies consider them a vulnerable population and found higher rates of mental health issues. This review aims to analyze the mental health status of the Roma population in Spain through existing literature. As a secondary objective, we have considered studying the degree of development of this research field in the Spanish context.

**Design:**

Due to the lack of clarity about the availability and quality of the existing information, a scoping review on the mental health status of the Roma population in Spain was performed. The databases included Medline, PsycInfo, Google Scholar, and Dialnet, covering the period 1993–2023. Experimental or quasi-experimental studies and observational studies, cohort studies, case-controls, cross-sectional studies, and case series were selected using PRISMA methodology. The results were analyzed from inductive thematic categorization.

**Results:**

Higher rates of depression and anxiety have been reported in Roma Spanish people, while drug and alcohol use might be increased in Roma men. The study of other psychiatric and psychological conditions is limited and biased. Some protective sociocultural factors have also been described such as ethnic identity, community self-esteem, or social organization based on the extended family. The research methodologies applied are heterogeneous in approach and sampling and deficient in experimental studies. The variability in topics and study designs makes the results neither replicable nor comparable.

**Conclusions:**

The mental health of the Spanish Roma population has been scarcely and vaguely studied. Preliminary research points at a worse mental health state in some areas but the field needs further development. There is also a lack of quality and standardization in the studies, and there is also a need for greater involvement of the Roma community itself. Research on the mental health of the Roma population in Spain should be perceived as a necessity and an opportunity for managers and professionals of health services and social services.

## Introduction

The Roma population represents the largest ethnic minority in Spain and Europe. As Filigrana [1] highlights, historical evidence suggests that their origins can be traced to a migratory movement from Northeast India around the tenth century. In Spain, the map on housing and Roma community [2] estimates the Roma population at 516,862 individuals, focusing on residents in neighborhoods with a high concentration of Roma people. However, broader studies by the Foundation for the Promotion of Social Studies and Applied Sociology [3] place the figure between 800,000 and 1,500,000 individuals.

The Roma population in Spain faces significant challenges in terms of mental health, as reflected in various studies and reports. A higher prevalence of mental health disorders, such as anxiety, depression, and substance abuse [4–6], has been observed, particularly among Roma women, who exhibit depression rates of 17.6%, compared to 7.7% in the general population [7]. However, it is striking the lack of research on other mental disorders and psychological conditions in this population.

Mental health disparities in ethnic minority populations are a global concern, with the Roma population being one of the most marginalized and underserved groups in Europe, including Spain. The Roma, historically subjected to social exclusion, discrimination, and economic marginalization, exhibit a range of health inequities, particularly in the domain of mental health. The Ministry of Social Rights, Consumption, and Agenda 2030 [8] has reported that 92% of Roma people in Spain are at risk of poverty or social exclusion, compared to 29.3% of the general population. According to a recent report by the University of Seville, the Roma are the largest ethnic minority group in Europe, often experiencing social exclusion. Social conflicts and limited access to healthcare are associated with increased psychopathological symptoms within this group [9]. Poverty, coupled with socioeconomic inequality [10, 11], exacerbates mental health vulnerabilities. Additional contributing factors include discrimination, antigypsyism, and disparities in education, housing, and access to healthcare, among others [12–14]. Antigypsyism remains a particularly persistent and socially accepted form of racism, complicating recognition and redress. Research shows a consistent association between experiences of discrimination and negative mental health outcomes, further deepening disparities [15–18]. These intersecting disadvantages collectively place the Roma community at a heightened risk of poor mental health outcomes [19]. A study conducted in Cantabria highlighted these social determinants of health among the Roma population contribute to the deterioration of their mental well-being [20].

The Roma population in Spain also faces numerous barriers to mental health care, including cultural stigma, underdiagnosis, and limited access to treatment. Mental health is often perceived as a private issue to be managed within the family, leading to delays in seeking professional help [21]. Furthermore, engaging with Roma communities, particularly nomadic groups, is complex and requires culturally sensitive approaches [22]. Much of the available information originates from NGO reports or public health collaborations rather than peer-reviewed studies. These factors limit both the volume and the quality of research in this field.

It is important to note that the scarcity of specific data on the mental health of the Roma population presents a barrier to the development of effective interventions. The “Fundación Secretariado Gitano” emphasizes the need for more detailed data collection to adequately address the health disparities faced by this community [7]. Despite existing studies that address mental health issues in the Roma population in Spain, further research and data collection are required to design targeted health policies and programs that effectively meet the specific needs of this group.

For this reason, we have chosen the format of an exploratory systematic review, which allows for a broad approach to fields of study where the evidence is diverse, scarce, and not well-systematized, such as the field of mental health in the Roma population in Spain. This study is the first attempt to review and provide a comprehensive overview of the field in Spain. It is expected that this review will be instrumental in shaping future research and interventions aimed at improving the mental health of the Roma population.

By synthesizing existing knowledge, it is expected that this review will be instrumental in shaping future research and interventions aimed at improving the mental health of the Roma population. Some of the gaps identified, which this review aims to clarify, are related to the type of psychopathological condition being studied. We know that depression, anxiety, and drug use have been prioritized over other areas of study, which not only makes the knowledge scarce but also biased. There is also a tendency to study mental health risk factors in the Roma population without considering potential protective factors, again implying a bias and prejudice toward this population. Another gap pointed out by preliminary studies concerns the methodological quality of the research. For this reason, the review not only analyzes the results related to mental health variables but also collects methodological variables from each of the selected studies.

Based on this preliminary observation, we have focused our research on two main objectives. On the one hand, we consider it essential to summarize and discuss the mental health status of the Roma population in Spain. On the other hand, we will delve into the level of development of this research field and the methodological quality of the studies.

## Materials and Methods

### Design

This study employed a systematic bibliographic review of an exploratory nature, commonly referred to as a “scoping review.” The limited volume and heterogeneity of the research on the mental health of the Roma population in Spain, as identified in our preliminary work, led us to choose an exploratory systematic review. Scoping reviews are useful for mapping the existing evidence in a broad or emerging field of research. They help identify gaps in knowledge, clarify concepts and definitions, and provide an overview of the scope of a topic. They are especially valuable when the research area is diverse or underexplored, making them an ideal precursor to more detailed systematic reviews.

### Objectives and Review Questions

This review aims to address two primary objectives: (1) mental health assessment: To evaluate the mental health status of the Roma population in Spain and identify disparities compared to the general population; (2) research development analysis: To assess the scope, quality, and methodologies of existing studies on Roma mental health in Spain.

We have posed several research questions to address these two issues, which include the main gaps and concerns detected. In relation to the first objective, we have asked ourselves: What is the overall mental health status of the Roma population in Spain? What variables have been studied in the field of their mental health, and which ones have been overlooked? Are there protective factors (and not just risk factors) for the mental health of Roma people in Spain? In relation to the second objective, we have asked ourselves: Do the identified studies have sufficient methodological quality? Is there standardization of the instruments used? What types of samples are studied, and which ones are not? To what extent is the Roma community involved in the studies?

These and other issues that have emerged through a reasoned critique of the study’s results may contribute to the optimization of future research designs, the clarification and destigmatization of this field of study, and above all, to the clarification of the evidence, which enables the design of future lines not only of research but also of psychosocial intervention.

### Search Procedure

For the conduct of this review, we followed the PRISMA guidelines for scoping reviews. The PRISMA Checklist is attached and can be consulted in Appendix 2.

A preliminary search in four databases (Medline, PsycInfo, Google Scholar, and Dialnet) led to the identification of the most commonly used keywords in this field of research. MeSH terms were used for Medline, Dialnet, Google Scholar, and PsychInfor, and an additional search following APA Thesaurus terms was carried out for PsycINFO. After this preliminary search, a search was conducted in each of the four databases. Medline is the main biomedical database. PsychINFO is a specialized database for psychology and mental health. Meanwhile, DIALNET contributes with its wealth of articles from Spanish sources. Finally, Google Scholar broadens the search scope due to its vast coverage and lower field specificity, promoting the detection of grey literature.

Systematic searches were conducted in Medline, PsycInfo, Google Scholar, and Dialnet databases between October 2023 and January 2024. The review covered a 30-year period, from 1993 to November 2023, with searches conducted in both English and Spanish. Boolean operators (“AND” and “OR”) and truncation symbols ([*]) were used to refine the search process, ensuring comprehensive inclusion of relevant variations in terminology.

Two independent searches were performed in each database for each language. The search strings used were:

- For searches in English language: “Mental Health” OR “Psych*” AND “Gypsy” OR “Gypsies” OR “Roma” OR “Romani” AND “Spain” OR “spanish”
- For searches in Spanish language: “Salud Mental” OR “Psic*” OR “Psiq*” AND “Gitan*”OR “Roma” OR “Romani” AND “Españ*”

Of these, the terms “Mental Health”, “Gypsy”, “Gypsies”, “Roma” and “Romani” were obtained by consulting MeSH terms—Ovid Medical Subject Headings. To improve specificity in Google Scholar, terms related to “Mental Health” and “Salud Mental” were restricted to title and abstract fields. A detailed account of the search strategy is provided in Appendix 1.

As we have stated, we conducted in PsychINFO a supplementary search using the APA Thesaurus term proposed by this database “Romanies.” To maximize the number of retrieved records, we used the search string “Romanies” and “Spain OR Spanish” considering that PsycInfo is already a specialized database.

Titles and abstracts were reviewed online, and full texts of studies meeting the inclusion criteria were obtained for further analysis. The screening and data extraction processes were conducted independently by two researchers (Lara Pais and Lucía del Río-Casanova). A medical faculty librarian advised authors 1 and 2 on the search strategy. For cross-reference detection, the bibliographic lists of each article that met the inclusion criteria were reviewed.

The review uses both “Gypsy” and “Roma” as terminological references to the population under study. While “Gypsy” can have pejorative connotations in some contexts [23], it has been resignified in Spain and is widely used both within the community [24] and in Spanish scientific literature. The term “Roma” is also used, in alignment with the Council of Europe’s 2012 definition [25], which encompasses a broad range of related groups, including Roma, Sinti, Kale, Travelers, and Eastern Dom and Lom communities, as well as individuals who self-identify as Roma.

The strengths of this search strategy include its systematic approach, replicability, and the use of both international and national databases, as well as databases of biomedical, psychological, and general scope which facilitates the detection of grey literature. As a limitation, we highlight that it has not been blindly verified by two different librarians.

### Screening and Selection

The inclusion criteria used were:

- Studies focused on the Roma population in Spain, regardless of whether it is any specific subgroup [settled, travelers, migrants, refugees…].
- Studies exploring mental health status, including both the psychiatric and psychological dimension.
- Studies that presented original data.

Thus, they were considered experimental (or quasi-experimental) and observational studies, whether these were analytical, descriptive, quantitative, or qualitative: clinical trials (randomized or not), cohort studies, case-controls, cross-sectional, case series.

As for the exclusion criteria, the discarded studies were:

- Studies that despite including Roma population, studied broader samples without segregating the results by ethnicity, so that the specific results for Roma population could not be extracted
- Opinion articles.

### Data Extraction

Data extraction was carried out independently by researchers 1 and 2. The information was systematically organized using the PICOS system (participants, interventions, comparisons, outcomes, study) and synthesized in tables as proposed by the PRISMA criteria for scoping reviews (PRISMA-ScR) [27].

## Results

### Search Results

**Fig. 1:**
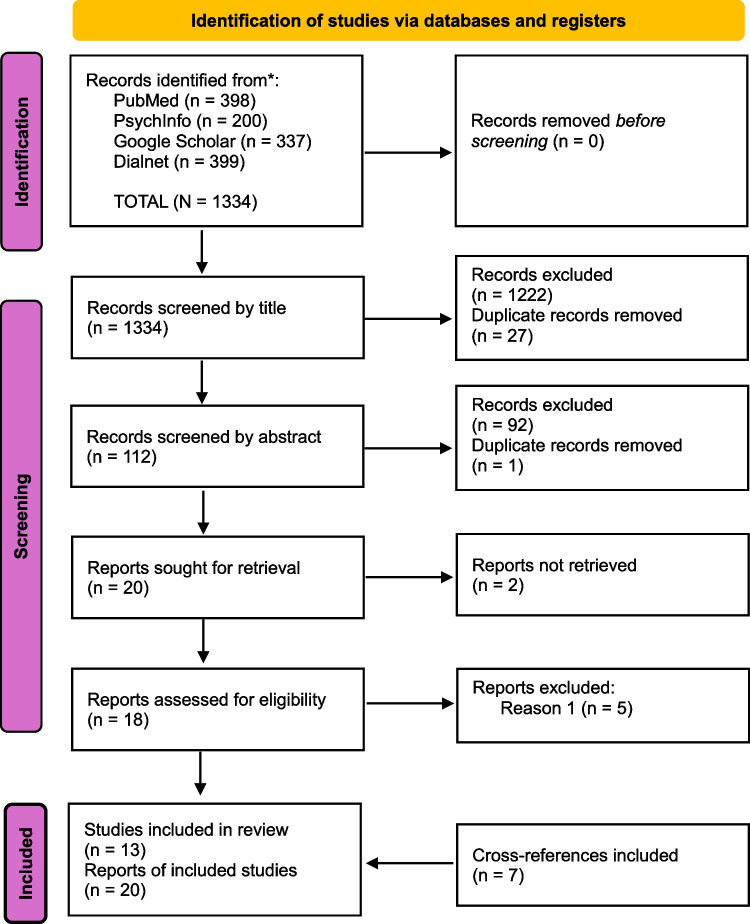
Flow chart of the identification and selection of studies process. Source: adapted from the PRISMA 2020 statement: an updated guideline for reporting systematic reviews [26]

A total of 1334 records were identified. An initial screening was conducted based on titles, including all entries referring to mental health, well-being, or general health focusing on the Roma population or minority groups they may belong to, to determine whether any psychological or psychiatric variables were examined. Figure 1 illustrates the identification and selection process. Most records were excluded at this stage, as they were either not focused on the Roma population, did not address health-related topics, or were cross-references unrelated to the research topic. A total of 139 entries were initially selected, 27 of which were duplicates.

After removing duplicates, 112 records remained for further abstract-based screening (PubMed *n* = 25, PsycInfo *n* = 34, Google Scholar *n* = 6, Dialnet *n* = 47). During this stage, the inclusion criteria described for the review were strictly applied. Articles that did not mention any exploratory variables related to mental health status were excluded, while those addressing perceived health and/or life satisfaction were retained. After identifying and excluding one additional duplicate, 20 articles were deemed eligible for full-text review. Of these, 2 were unobtainable, and 5 of the remaining 18 were excluded as they did not investigate any exploratory variables of mental health status.

During the full-text review, several cross-references of interest were identified. Of these, 8 met the inclusion criteria for this review, although one was excluded as its articles had already been analyzed independently in this work. Consequently, 7 cross-references were included. In total, this review considers 20 studies.

### Participants

Of the 20 total studies, 16 recruited samples, while 4 used existing survey data or registries. Recruitment methods varied significantly: 15 studies employed non-probabilistic methods (such as convenience, purposive, or quota sampling), 3 used probabilistic methods, and the remaining 2 did not specify their recruitment methods.

Table 1 details the number of Roma individuals in each sample, showing considerable variability. Only 3 studies calculated the required sample size.

**Table 1:** Summary of results: PICOS data collection

Title (author, year), origin	Participants	Interventions and instruments	Main comparisons	Key results: outcomes	Design, methodology, and sampling
Attitudes of adolescent Spanish Roma toward noninjecting drug use and risky sexual behavior (Garcia de Cortazar A et al. 2009) [30]	*N* = 37 Roma population. (men: 20, women: 17. Age (range): [16–17] [18–24] 17	Semi-structured ad hoc *interviews*, discussion groups	Sexual behavior under the influence of drugs. Opinions on interventions relating to drug use. Influence of gender	Adult males believe that cocaine helps them free themselves from a repressive sex education They question the message “Don’t consume” but value informational interventions and would like to expand them on the consequences of consumption Consumption declared lower in women than in Roma men	Qualitative and descriptive (cross-sectional) study Non-probability sampling (intentionally done by Roma associations)
What are Roma like and what do they suffer from? (Cabedo García VR et al. 2000) [32]	*N* = 121 Roma people of one health center. (men: 53, women: 68). Age (range): [14– ≥ 80]	Ad hoc *questionnaire* for review of medical records	Assessment of socio-health risk and prevalence of chronic diseases and their risk factors	Higher prevalence in the Roma population of socio-health risk (*p* < 0.01), excessive substance use (heroin, cocaine) (*p* < 0.01), and alcohol (*p* < 0.05) No significant differences in smoking	Mixed study: qualitative and quantitative, descriptive (cross-sectional) Sampling: non-probabilistic (convenience)
Cultural and Religious/Spiritual Beliefs and the Impact on Health that Fear to Death has on Gender and Age, Among a Romani Minority Group from Southern Spain (Restrepo-Madero E. et al. 2017) [44]	*N* = 150 people 95.6% Roma. men: 47.3%, female: 53.7%. Age (mean): 41.5 years (SD: 14,9)	Questionnaire: ESI (expressions of Spirituality Inventory), CL-FODS (Collett- Lester Fear of Death Scale), NHP (Nottingham Health Profile)	Perception of health and the incidence in it of religion, spirituality, fear of death, and paranormal beliefs	Negative impact: age, female gender, negative sense of life, fear of death from others, few paranormal beliefs. Results consistent with the general population, with the differences that in the Roma population, paranormal beliefs are protective, and fear of the death of others harms health to a greater extent than fear of death itself	Qualitative, descriptive (cross-sectional, exploratory) study Sampling: non-probabilistic (convenience)
Differences in the prevalence of depression in older Spanish Romany and non‐Romany people and associated factors (Heredia-Amador Á et al. 2018) [34]	*N* = 95 Roma people (men: 45, women: 50). Age (range): > 55 years old	Questionnaire GDS-EV (Geriatric Depression Scale score, Yesavage, Spanish version)	Risk of depression in relating to the general population	RR depression or probable depression = 4.2 in Roma men (*p* < 0.01) and 2.2 in Roma women (*p* < 0.01) Perception of being valued by the family. Greater protective effect of activity at home and physical exercise	Mixed study (qualitative and quantitative), descriptive and analytical Sampling: non-probabilistic (quota-based)
Drugs and Mental Health Problems among the Roma: Protective Factors Promoted by the Iglesia Evangelica Filadelfia (López J et al. 2018) [50]	*N* = 9 Roma people Men: 3 Women: 6 Age (range): 25–60 years. Age (mean): 41.2	Communicative everyday life stories (CELS)	Factors promoting rehabilitation, protective against drug use, and facing mental health problems	Anti-drug discourse, support environment, model roles, new social relationships, promotion of welfare/happiness discourse, sense of belonging, beliefs against suicide	Qualitative, descriptive study (communicative methodology) Sampling: non-probabilistic (intentional)
Effects of a dialogue-based program to improve emotion knowledge in Spanish Roma preschoolers (Giménez-Dasí M et al. 2017) [42]	*N* = 43 Male: 17 Female: 26 Age (range): 4–5 years	Program of Intervention in emotional intelligence “Thinking Emotions,” (Giménez-Dasí et al. 2013), based on dialogue between peers	ECT, AKT, VAVEL, and CEIC	After intervention, the experimental group improved significantly in the causality and regulation components of TEC and the AKT variables related to atypical causality. Significant improvement in TEC and AKT when language covariable was introduced. Moderately high but not significant effect on CEIC	Quantitative study analytic (quasi-experimental) Sampling: non-probabilistic (convenience)
Gypsies and Drug Addictions: Study of Adherence to Treatment (Iraurgi I et al. 2000) [31]	*N* = 52 Roma men: 3.1% Female: 26.9% Age (mean): 25.1 years, (SD: 4.2)	Add hoc questionnaire	Review of consumption and permanence variables	The characteristics of consumption do not differ substantially, except in the lack of prevention measures. In total, 100% of the women in the sample are married couples of addicted men. The continuity rates of the Roma population are lower for the entire period studied, but not significant (*p* = 0.07)	This is a mixed, descriptive and analytical study (retrospective cohort) Sampling: non-probabilistic (convenience)
Health status of Roma women in Spain (Carrasco-Garrido P. et al. 2011) [35]	*N* = 527 Roma women. Age (range): > 16 years old. Age (mean): 36.3 years	“National Health Survey of Spain 2006” (INE) “National Health Survey of the Roma Population 2006” (MSyC, FSG)	Diagnosed health problems, lifestyle Compared to women in the general Spanish population	Roma women are more likely to be diagnosed with depression/anxiety (OR = 1.95) than non-Roma. In addition, higher values of alcohol use (OR = 3.8), if lower for tobacco (OR = 1.9)	Mixed study (qualitative and quantitative), descriptive (epidemiological transverse) Sampling: Multi-ethnic probabilistic
Incidence of infectious diseases and survival among the Roma population: a longitudinal cohort study (Casals M et al. 2012)	*N* = 380 Roma people Men: 192 Women: 188 Age (median): 14.6 years	Ad hoc *questionnaires* collected in an outbreak of tuberculosis, population census and other records	Use of injectable drugs (IDU) and type of drug	High incidence rates of IDU in the Roma cohort (240 cases/100,000 pyf) compared to the general population of Barcelona during the same period (1987–2008)	This is a descriptive study (retrospective cohorts) Sampling: No Probabilistic (for convenience)
Abused Roma children: socio-health risk factors and priority health needs (Oliván Gonzalvo G 2004) [46]	N = 74 Roma in child protection centers Men: 49.7% Women 50.3% Age (mean): 6.9 years (DE: 5.7)	Review of socio-sanitary files	Prevalence of abuse factors of socio-sanitary risk in the family. Psychomotor development Conduct disorders	Greater and significant prevalences in: passive abuse (OR = 2.4), belonging to families with more than one socio-health risk factor (OR = 30.5), crime problems (OR = 11.7), drug/alcohol abuse (OR = 3.4), delayed psychomotor development in children under 6 (OR = 2.4), and behavioral disorders in adolescents (OR = 4.7)	This is a mixed, descriptive, and analytical study (retrospective and cross-sectional cohort) Sampling: non-probabilistic (convenience)
Roma Never Die Alone (García-Espinel T et al. 2017) [39]	*N* = 1 Roma woman 4 family and community members and 2 officers	2 communications according to the method of communication and observation (COM) 6 communicative daily life stories (CDLS)	Roma community values related to end-of-life and possible benefits of these for patients	Health institutions designed for different people, perceiving the misfit, misunderstood and infantilized proposals: flexibility for customs and sensitivity to the pain of friends and family (recognizing the pastor’s figure). Seniors as mediators to reduce distances. Consider the diversity of the population	Qualitative, descriptive study (communicative methodology) Sampling: non-probabilistic (intentional)
Roma women’s perspectives on end-of-life decisions (Peinado-Gorlat P et al. 2015) [38]	*N* = 33 female Roma caregivers Age (mean): 38.6 years	Discussion groups	Role of family, preferences in care, and treatment Relationship with the health system	Great influence of community opinion on personal or family decisions. Everyone believes the home is the ideal place to serve their family members “it is the family who knows what is best for a patient” Ignorance and rejection of the document	This is a descriptive study Sampling: non-probabilistic (intentional and for convenience)
Scripts or Components? A Comparative Study of Basic Emotion Knowledge in Roma and Non-Roma Children (Giménez-Dasí M et al. 2018) [41]	*N* = 47 Roma people schooled in a concerted school (men: 17, women: 30) Age (range): 4–5 years	Simple semi-structured ad hoc *interview*, with open response	Knowledge of basic emotions in children. Comparison with a non-Roma	Significant differences in TEC (marked in identification, causality, and emotion regulation) and vocabulary testing. The emotion that the general population defined most complexly was anger (31%), while for Roma boys and girls, it was fear (21.6%), and they showed a significant lack of definition about happiness (20.7%)	This is a mixed, descriptive and analytical (cross-sectional) study Sampling: non-probabilistic (convenience)
The health status of delinquent gipsy youths in Spain (Oliván Gonzalvo G 2002) [5]	*N* = 222 Roma criminals (men: 92%, women: 8%) Age (range): 13–19 years	Review of clinical and social histories	Health status and acute or chronic diseases	Similar “health problems” with a “slightly higher” smoking and drug abuse rate	Descriptive study (retrospective cohort) Sampling: non-probabilistic (convenience)
The role of group identification in the well- being of Spaniards with gypsy ethnicity (Gómez-Berrocal M et al. 2020) [40]	*N* = 229 Roma (men: 44.5%, women: 55.5%) Age (range): 18–89 years. Age (mean): 36.57 years (SD = 15.2)	Survey. SPWB e SWLS (Psychological Wellbeing and Life Satisfaction); MEIM (Multigroup Ethnic Identity Measure); SIO (self-ingroup overlap)	Psychological well-being and life satisfaction Ethnic identity and sense of belonging to the group	The level of studies relates significantly to life satisfaction but not psychological well-being, with which objective conditions of deprivation are also not associated Participants with higher scores in ethnic identity referred to significantly greater well-being and life satisfaction The feeling of ethnic belonging affects more areas of well-being than proximity to the group	Mixed study (qualitative and quantitative), descriptive and analytical (cross-sectional) Non-probability sampling (convenience)

Sources: own elaboration

All studies focus specifically on the Spanish Roma population, with 12 comparing it to the general Spanish population. Most studies are localized (only 5 collect data across the country), which introduces selection bias and reduces the external validity of the findings for the broader Roma population in Spain.

Regarding sample characteristics: 13 studies focus on the adult population overall, 4 studies focus on childhood or adolescence, 1 study targets youth (ages 16–24) and 2 studies examine individuals over 55 years old. Some studies are conducted in specific subgroups, such as members of churches, youth involved in criminal activities, minors in protective care, patients undergoing maintenance programs with naltrexone, or female caregivers of family members.

A total of 17 studies include both genders or do not specify gender, while 3 studies focus exclusively on women. Roma women represent approximately 55% of the participants. Age is reported in a heterogeneous manner and mean age was not calculable. All age ranges are represented in the studies, but with varying weights. Only 2 studies include participants under age 13.

### Interventions

The most common methods were interviews and/or discussion groups (*n* = 6), whether structured, semi-structured, or exploratory, and self-reported data collection (*n* = 6), which included 3 questionnaires, 2 communicative accounts of everyday life, and 1 quasi-experimental approach. Retrospective analysis using records from existing databases (*n* = 4) was also prevalent. Some studies relied primarily on various National Health Surveys related to the Roma population (*n* = 3). Others used methods that were insufficiently specified (*n* = 2, expert reports). Most studies are conducted by non-Roma researchers.

### Comparisons and Outcomes

Summary tables were created to present the results. Table 1 includes research articles published in scientific journals, while Table 2 compiles other works such as reports and doctoral theses. Following the selection process, we proceeded to read and analyze the publications guided by the PRISMA Extension for Scoping Reviews (PRISMA-ScR) criteria [27]. The results were analyzed through a descriptive synthesis and an inductive analysis of content, organized into topics and subtopics [28]. Additionally, the recommendations regarding the structure and methodology of each study were considered based on the guidelines provided by the Joanna Briggs Institute [29].

**Table 2:** Summary of results (grey literature)

Article: title, author, year, participants	Key results
The impact of the crisis on the Roma community. (FSG Report, 2013) *N*: not described	Increased mental health problems due to stress, depression, or anxiety, especially in Roma women
Towards equity in health: A comparative study of national health surveys of the Roma population and the general population of Spain. (La Parra Casado D, 2006). *N* = 1500	Depression plus men (7.5% vs 2.9% overall) and Roma women (17.6% vs 7.7%) They refer to “other mental illnesses” plus men (4.8% vs 1.9% overall) and Roma women (5.9% vs 2%) Fewer Abstemian Roma men (24.8% vs 31.3% overall) and more Roma women (61.2% vs 55.9%). More Gypsy smokers (54.9% vs 31.6% overall), especially in 16–24 years and lower educational level. The number of people sleeping 5 or fewer hours a day exceeds the general population
“National Health Survey 2006–2007” (ENSE 2006). National Institute of Statistics (INE), MSyC	More tranquilizers, relaxants or sleeping pills in men (11.7% vs 4.7% overall) and Roma women (15.6% vs 11.6% overall). More antidepressants and/or stimulants in men (5.3% vs 1.7% overall) and Roma women (7.6% vs 4.3% overall). Consumption with higher prescription in the Roma population for all drugs
Roma old age: a psychoanthropological study of cultural differences in ageing processes and their psychosocial consequences. (Heredia-Amador Á, 2018). PhD Thesis. *N* = 95 Roma > 55	4 times more depressed than non-Roma, with a greater difference in women (6 vs 2) that tends to match age. With GDS-ES fewer cases, with nearly 3 times as many Roma population at risk. Subclinical depression was far higher in the Roma group (up to 46%). Almost 7% of Roma men are smokers, women smoke 3 times less. Lower stated consumption of alcohol in the Roma population
Health and the Roma community. (MSyC, FSG. 2005). *N*: not described	In Roma women: symptoms of depression, distress and anxiety about the traditional role, early abandonment of mental health treatments. Protective factors: cultural identity, pride and community self-esteem vs individuality. The extended family offers safety, resources and care but it can bring negative consequences if the life projects you point out are very rigid. Intense relationship with the transcendental
Second National Health Survey of the Roma Population 2014. Report of the Ministry of Health, Social Services and Equality. Spain. *N* = 1167 Roma people	“Depression” plus Roma men (5.4% vs 4.4% overall), fewer women (9% vs 10.6% overall). Refer to “other mental health problems” plus Roma men (3.3% vs 1.7% overall) and women (4.2% vs 1.4% overall). Smoke daily more Roma men (54.1% vs 28.3% overall) and fewer women (16.7% vs 21.7). Lower alcohol consumption in the Roma population for the last 2 weeks, but higher for the last 12 months in Roma men. Much more frequent intensive consumption episodes. More abstemious with age, especially in women

Sources: own elaboration

Main outcomes are presented in PICOS format with the data of the participants [P], interventions and instruments [I], comparisons [C], main results [O], and design [S] of two studies analyzed (Table 1; see in the “Results” section). For thesis, books, and reports, we elaborate a simplified data table (Table 2; see in the “Results” section]. The most relevant information was extracted and synthesized according to the following categories: participants and recruitment, methodology, and main results.

Only one study focuses on the overall mental health of the Roma population, examining the role of the Philadelphia Evangelical Church as a protective or rehabilitative factor in mental health issues and drug use [30]. The remaining selected studies can be classified into the following categories:

1. Studies on general health and mental status: These include research on overall health (*n* = 1) and mental health variables such as drug use (*n* = 4), alcohol use (*n* = 4), tobacco use (*n* = 5), and anxiety (*n* = 5) and depression (*n* = 5).
2. Studies on psychiatric mental disorders: These focus specifically on psychiatric disorders (*n* = 3), including alcohol and drug abuse (*n* = 2) and depression (*n* = 1).
3. Studies on psychosocial variables and mental health: This category includes research on psychosocial factors and mental health (*n* = 8), such as beliefs about death and grieving (*n* = 2), aging (*n* = 1), emotional intelligence in preschoolers (*n* = 2), religion, spirituality, and paranormal beliefs (*n* = 1), child abuse (*n* = 1), and ethnic identity and sense of belonging (*n* = 1).

Drug use is the most frequently studied topic (*n* = 8), with all studies indicating a higher prevalence in the Roma population. Women reported lower consumption compared to men, though cases of tranquilizer abuse were noted [30]. Additionally, all women participating in a maintenance program with naltrexone were partners of men who used drugs [31]. Roma men and women also exhibited higher consumption of tranquilizers, antidepressants, and/or stimulants compared to the general population [32, 33]. The protective role of the Church or “worship” was highlighted in two studies.

Seven studies addressed alcohol consumption, with almost all showing a higher prevalence in the Roma population, characterized by more frequent episodes of intensive drinking and an earlier age of onset. Significant differences were found among younger men [32]. Some studies noted a higher rate of abstinent Roma women compared to the general population [33, 34], while another study found higher alcohol consumption rates among Roma women [35].

Seven studies reported on depression and/or anxiety. All studies agreed on a higher prevalence and/or risk of depression in the Roma population, particularly among women. Roma individuals scored higher on variables associated with depression risk, with minimal correlation to income level or feeling valued by family members [36]. Anxiety risk is notably higher among Roma women [35]. Two reports mentioned increased stress and responsibilities due to dire economic, housing, and social conditions, with women often abandoning treatments prematurely. “Other mental illnesses” were also reported more frequently among Roma men and women compared to the general population.

Six studies investigated tobacco use, yielding heterogeneous findings. One study found no significant differences [32], while three studies noted a lower age of onset and a higher percentage of Roma male smokers, but a lower percentage of Roma female smokers [33, 36, 37].

Other topics covered include:

- End-of-life decision-making (*n* = 3): The family and community, especially women, play an important role, while the role of health technicians is respected but secondary. Care and solidarity are fundamental values, and disease is understood as a collective issue. They want the patient’s preferences to be considered, but not through a document that cannot be adapted to the circumstances [38]. It is perceived that health institutions are designed for people with different needs, which leads the Roma population to feel maladjusted, misunderstood, and infantilized [39].
- Life satisfaction and psychological well-being (*n* = 2): Nearly 65% more Gypsies than non-Gypsies report being “little” or “not at all” satisfied, with Roma women standing out negatively in this regard [34]. Life satisfaction is associated with educational level and ethnic identity, while psychological well-being is only associated with ethnic identity (not with educational level or objective deprivation). Ethnic identity has a protective role, stronger even than affinity with the social group [40].
- Self-perception of being valued by the family (*n* = 1): Fifteen percent more of the Roma population feel “very” or “quite valued” and receive frequent visits compared to the general population. Ninety-nine percent of the Roma population would not take their elderly, physically disabled, or mentally ill relatives to a care home, while 20% of the general population would. The social organization based on the extended family often provides resources and life plans for its members, which can act as a protection factor but may have negative consequences if they become too rigid [34].
- Attitude toward old age (*n* = 1): Older Gypsies tend to feel less useful and adopt a passive attitude of rest, resignation, and waiting, seeing this stage as close to death. At the same time, they take on a more active role in community participation and decision-making [39].
- Emotional education in preschoolers (*n* = 2): Gypsy children score significantly lower on the Test of Emotion Comprehension and vocabulary and have more difficulty defining emotions like fear and happiness [41] but show significant improvement under an intervention program [42].
- Impact of religion and spirituality on health (*n* = 2): There is a strong relationship between religion/spirituality and health in the Roma population [43]. Paranormal beliefs act as protectors, and the fear of a family member’s death harms health more than the fear of one’s own death [44]. Participation in the Church influences social dynamics in the Roma community, promoting collective identity, community support, conflict resolution, social cohesion, and women’s participation [43]. It also facilitates role models and social relationships, contributing to a sense of belonging. The Church itself promotes a discourse that bans consumption [43, 45], advocates for welfare and happiness, and opposes suicide [45].
- Sleep hours (*n* = 1): Roma men sleep more than 30 min longer than non-Roma men (with no significant difference for boys), while Roma women sleep more than 15 min longer than non-Roma women (but less than 15 min longer for girls). In contrast, among those over the age of 55, more Roma men and women sleep 5 or fewer hours a day [33].
- Child abuse (*n* = 1): The prevalence of Roma children admitted to protective facilities was 1.7 times higher, with a higher prevalence of passive abuse overall. Active abuse was more common among adolescent women, typically perpetrated by their husband or partner. The Roma population shows higher rates of psychomotor development delays in children, greater incidence of behavioral disorders in adolescents, and an increased risk of belonging to families at socio-health risk or with criminal problems [46].

### Study

Except for one quasi-experimental study, the remaining 19 were observational (descriptive methods *n* = 12, analytical methods *n* = 0, mixed methods *n* = 7, reviews *n* = 0). In total, 3 were quantitative, 7 qualitative, and the remaining 10 mixed-methods studies.

In 12 of the papers, the researchers used ad hoc questionnaires or interviews, while 8 included some standardized health or psychometric instruments validated in the Spanish population (in some cases, short versions). No study used instruments specifically validated for the Roma population.

In 7 of the papers, the presentation of mental health data was purely qualitative and descriptive. Of the remaining 13, 5 used descriptive statistical analysis, and 8 provided inferential statistical analysis. Among the latter, all performed some form of comparison test between groups, 5 also conducted tests of association between different variables (such as calculating correlation coefficients, relative risks, and probability ratios), and 2 elaborated regression analyses.

The studies are characterized by significant heterogeneity in their approaches, designs, and scientific rigor. The lack of experimental studies that allow for the rigorous development and evaluation of specific mental health interventions for the Roma population should be highlighted, with only one quasi-experimental study [42]. There is also heterogeneity in the size and characteristics of the samples across the studies.

## Discussion

### Summary of Evidence

The results confirm that the Roma population continues to experience worse health outcomes compared to the general population, with higher prevalence of depressive and anxiety disorders, as well as increased rates of smoking, drug use, and alcohol consumption. These findings are consistent with previous studies on the Roma population in other countries [32, 47, 48].

As in other European countries, higher rates of conduct disorders, developmental disorders, and antisocial behaviors have been reported in Spain. However, there are many other psychological disorders or situations of psychological distress that have not been detected in the studies found in Spain. In our opinion, it is notable the lack of research on issues such as post-traumatic stress (given the characteristics of the population) and other related pathologies. Specific studies on psychosis, personality disorders and suicidal behaviors have not been found. Other European countries have reported increased suicidal rates, so this issue should be addressed as soon as possible [49]. The rejection and difficulty in discussing this topic have been confirmed in the Spanish population.

The reviewed literature also identifies several mental health protective factors within the Roma community that counteract the multiple risk factors they face. Mental health protective factors are often overlooked in research, yet they are crucial when designing preventive interventions. These factors include:

- Cultural identity and community pride**:** A strong sense of cultural identity, community pride, and self-esteem, as opposed to a focus on individuality, serves as a protective factor [43]. Ethnic identity is linked to greater well-being and life satisfaction [40].
- Social organization and extended family**:** The extended family plays a crucial role, particularly for Roma women, serving as a source of resources and care. This collective approach to health issues, where individual ailments are viewed as communal problems, underscores the values of care and solidarity in Roma culture [38, 39]. This is exemplified by the lower rates of Roma individuals admitted to social care centers for dependency, disabilities, or mental illness [34, 43], and a higher perception of being valued by family. However, the view of aging among the Roma is often described as more negative, with greater feelings of worthlessness and a tendency towards resignation [34], though active participation and decision-making roles for seniors in the community are also noted [39].
- Transcendental and paranormal beliefs**:** Relationships with transcendental and paranormal beliefs [44], along with the church’s influence on community life, social cohesion, and a sense of belonging, also act as protective factors [43, 45, 50]. Traditional discourses, including church teachings against substance use and promoting well-being and happiness, are seen as potential protective elements. However, strategies that emphasize internal control and reduce reliance on external moralizing or rigid discourses might be more beneficial.

### Discussion with Results

From a gender perspective, the Gitano Secretariat Foundation [43, 51] highlights the burden of responsibilities and early abandonment of mental health treatments among Roma women. Gender differences in depression and substance use rates identified in this review are also documented in international literature [52, 53]. These reports emphasize the need for progress in domestic and family co-responsibility for Roma men, as well as improvements in their health management and prevention of accidents and substance use.

Additionally, the influence of prejudices against the Roma population should be considered, as they can impact research and health services in various ways [54]. Prejudices may shift the focus from the health status of minorities, such as the Roma, to concerns about the broader social majority, as highlighted by [55]. Stereotypes about social groups can shape expectations and interactions through subtle and often unconscious processes, increasing the likelihood of discrimination and lower-quality service from health and research professionals.

The poor mental health outcomes observed in Spanish Roma are consistent with previous research on poverty and socio-economic inequity [10, 56], social exclusion, and ethnic minorities [57]. Additionally, variability in the manifestation and interpretation of diseases [58], as well as the effects of discrimination [59] and racism [60, 61], may contribute to these outcomes.

Finally, a low level of involvement from the Roma community in research has been detected. The principal researchers are from outside the community, and although there are occasions when community leaders are involved, sometimes this figure is excluded, and in other cases, their role is limited. One might wonder to what extent this is related to the limited access to higher education and, therefore, to the research career of the Roma population, and to what extent the interest (or lack thereof) in mental health as a research topic within this ethnic group influences this situation.

### Practical Implications

Additionally, the lack of Roma researchers in designing and conducting studies on Roma health leads to biases and research questions that may not align with the community’s needs. Most studies are conducted by non-Roma researchers, which can result in misaligned research questions and findings. Communicative methodologies that break down traditional hierarchies between researchers and subjects, prioritizing group-based reflection and value enhancement without replacing them, can address these issues [39, 42]. Furthermore, using dialogue with healthcare personnel to better express cultural singularities in health spaces may improve access to health services for the Roma community [39]. Community-based participatory research-action methodologies could also be valuable, enabling Roma communities to advocate for their health, generate relevant knowledge, and identify necessary support and resources [62].

Finally, from a methodological point of view, this review shows that the challenges lie in conducting specific, high-quality research with scientifically validated methods and instruments, profiles of more socially and geographically diverse Roma populations, and diverse age ranges, psychosocial pathologies, health statuses, and gender diversity. Multidisciplinary research is also needed to address issues related to data privacy, inequality in living conditions, housing, education, access to technology, energy availability, and access to service networks. Considering the benefits of research for the care of Roma people, and addressing the barriers they face, it could motivate organizations providing health and social services to become more effective.

### Limitations

This scoping review is the only one specifically focused on mental health in the Roma population in Spain, providing a comprehensive examination of the available literature. The exploratory nature of this study allowed us to include grey literature and employ mixed methodologies (both qualitative and quantitative), facilitating a broad perspective on this neglected research field.

However, there are some limitations to consider. The review adopts a psychosocial perspective but does not incorporate anthropological or sociological viewpoints, nor does it include databases from these fields. Due to the heterogeneity of the included studies, the results were analyzed descriptively and qualitatively. Moreover, not all relevant knowledge may be covered by the types of sources reviewed, and a significant portion of the included works were cross-references rather than primary findings from the search itself. Finally, it is important to highlight that most of the research, including this review, was conducted by non-Roma researchers, which may introduce biases and limit the applicability of the findings to the conclusions.

## Conclusions

This scoping review highlights that research on mental health in the Roma population in Spain is limited. The most studied topics are drug, alcohol, and tobacco use, reflecting the perception of higher consumption rates in this population. Higher prevalence of depressive and anxiety disorders was also observed. However, there is a notable lack of study on other psychopathological disorders, such as psychotic disorders, suicidal behavior, traumatic stress disorders, and personality disorders.

Protective factors in the Roma community, such as cultural identity, social organization, and transcendental beliefs, seem to counteract the risks associated with social exclusion and adverse socio-economic conditions. Despite these strengths, research on the mental health of the Roma population needs to improve in terms of quality, methodology, and population diversity.

In general, future research should incorporate participatory and collaborative approaches with the Roma community, fostering the development of specific interventions that address their mental health needs. Moreover, limitations related to data privacy, living conditions, and access to services should be considered.

## Appendix 1

see Table 3

**Table 3:** Final search strategy

Databases	Search dates	Search strings	Filters applied
Medline	Oct 2023– Jan 2024	“Mental Health” OR “Psych*” AND “Gypsy” OR “Gypsies” OR “Roma” OR “Romani” AND “Spain” OR “spanish”	Date filters: 1993–Nov 2023 Language filters: English, Spanish
	Oct 2023–Jan 2024	“Salud Mental” OR “Psic*” OR “Psiq*” AND “Gitan*” OR “Roma” OR “Romani” AND “Españ*”	
PsycInfo	Oct 2023–Jan 2024	“Mental Health” OR “Psych*” AND “Gypsy” OR “Gypsies” OR “Roma” OR “Romani” AND “Spain” OR “spanish”	
	Oct 2023–Jan 2024	“Salud Mental” OR “Psic*” OR “Psiq*” AND “Gitan*” OR “Roma” OR “Romani” AND “Españ*”	
Google Scholar	Oct 2023–Jan 2024	“Mental Health” OR “Psych*” AND “Gypsy” OR “Gypsies” OR “Roma” OR “Romani” AND “Spain” OR “spanish”	
	Oct 2023–Jan 2024	“Salud Mental” OR “Psic*” OR “Psiq*” AND “Gitan*” OR “Roma” OR “Romani” AND “Españ*”	
Dialnet	Oct 2023–Jan 2024	“Mental Health” OR “Psych*” AND “Gypsy” OR “Gypsies” OR “Roma” OR “Romani” AND “Spain” OR “spanish”	
	Oct 2023–Jan 2024	“Salud Mental” OR “Psic*” OR “Psiq*” AND “Gitan*” OR “Roma” OR “Romani” AND “Españ*”	

Source: own elaboration

## Appendix 2. PRISMA checklist

**Table:** 

	Item	Prisma-ScR checklist item	Reported on page #
Title			
Title	1	Identify the report as a scoping review	1
Abstract			
Structured summary	2	Provide a structured summary that includes (as applicable): background, objectives, eligibility criteria, sources of evidence, charting methods, results, and conclusions that relate to the review questions and objectives	1
Introduction			
Rationale	3	Describe the rationale for the review in the context of what is already known. Explain why the review questions/objectives lend themselves to a scoping review approach	2
Objectives	4	Provide an explicit statement of the questions and objectives being addressed with reference to their key elements (e.g., population or participants, concepts, and context) or other relevant key elements used to conceptualize the review questions and/or objectives	2
Methods			
Protocol and registration	5	Indicate whether a review protocol exists; state if and where it can be accessed (e.g., a Web address); and if available, provide registration information, including the registration number	3
Eligibility criteria	6	Specify characteristics of the sources of evidence used as eligibility criteria (e.g., years considered, language, and publication status), and provide a rationale	3
Information sources*	7	Describe all information sources in the search (e.g., databases with dates of coverage and contact with authors to identify additional sources), as well as the date the most recent search was executed	3
Search	8	Present the full electronic search strategy for at least 1 database, including any limits used, such that it could be repeated	Appendix A
Selection of sources of evidence†	9	State the process for selecting sources of evidence (i.e., screening and eligibility) included in the scoping review	3,4
Data charting process‡	10	Describe the methods of charting data from the included sources of evidence (e.g., calibrated forms or forms that have been tested by the team before their use, and whether data charting was done independently or in duplicate) and any processes for obtaining and confirming data from investigators	3, 4
Data items	11	List and define all variables for which data were sought and any assumptions and simplifications made	3, 4
Critical appraisal of individual sources of evidence§	12	If done, provide a rationale for conducting a critical appraisal of included sources of evidence; describe the methods used and how this information was used in any data synthesis (if appropriate)	NA
Synthesis of results	13	Describe the methods of handling and summarizing the data that were charted	3, 4
Results			
Selection of sources of evidence	14	Give numbers of sources of evidence screened, assessed for eligibility, and included in the review, with reasons for exclusions at each stage, ideally using a flow diagram	4, 5
Characteristics of sources of evidence	15	For each source of evidence, present characteristics for which data were charted and provide the citations	5, 6
Critical appraisal within sources of evidence	16	If done, present data on critical appraisal of included sources of evidence (see item 12)	NA
Results of individual sources of evidence	17	For each included source of evidence, present the relevant data that were charted that relate to the review questions and objectives	5–7
Synthesis of results	18	Summarize and/or present the charting results as they relate to the review questions and objectives	7, 8 16–21
Discussion			
Summary of evidence	19	Summarize the main results (including an overview of concepts, themes, and types of evidence available), link to the review questions and objectives, and consider the relevance to key groups	8, 9
Limitations	20	Discuss the limitations of the scoping review process	9, 10
Conclusions	21	Provide a general interpretation of the results with respect to the review questions and objectives, as well as potential implications and/or next steps	11
Funding			
Funding	22	Describe sources of funding for the included sources of evidence, as well as sources of funding for the scoping review. Describe the role of the funders of the scoping review	11

## Data Availability

Not applicable.

## Code Availability

00–02.
